# Epithelial-to-Mesenchymal Transition-Related Markers in Prostate Cancer: From Bench to Bedside

**DOI:** 10.3390/cancers15082309

**Published:** 2023-04-14

**Authors:** Samantha Gogola, Michael Rejzer, Hisham F. Bahmad, Wassim Abou-Kheir, Yumna Omarzai, Robert Poppiti

**Affiliations:** 1 Herbert Wertheim College of Medicine, Florida International University, Miami, FL 33199, USA; sgogo002@med.fiu.edu (S.G.); mrejz001@med.fiu.edu (M.R.); 2 The Arkadi M. Rywlin M.D. Department of Pathology and Laboratory Medicine, Mount Sinai Medical Center, Miami Beach, FL 33140, USA; yumna.omarzai@msmc.com (Y.O.); robert.poppiti@msmc.com (R.P.); 3 Department of Anatomy, Cell Biology and Physiological Sciences, Faculty of Medicine, American University of Beirut, Beirut 1107, Lebanon; wa12@aub.edu.lb; 4 Department of Pathology, Herbert Wertheim College of Medicine, Florida International University, Miami, FL 33199, USA

**Keywords:** prostate cancer, epithelial-to-mesenchymal transition, mesenchymal-to-epithelial transition, EMT, MET, biomarkers, targeted therapy, review

## Abstract

**Simple Summary:**

Prostate cancer (PCa) is the second most frequent type of cancer in men worldwide. Treatment options for early-stage PCa include external beam radiation therapy, brachytherapy, radical prostatectomy, active surveillance, or a combination of these. In most patients, however, PCa eventually progresses to castration-resistant prostate cancer (CRPC). Transition of PCa from an androgen-dependent to androgen-independent state is not yet fully understood, but epithelial-to-non-epithelial (“mesenchymal”) transition (EMT) plays a crucial role in this process. In this review, we provide a synopsis of the transcriptional factors and signaling pathways involved in EMT, besides the diagnostic and prognostic biomarkers that have been identified in this process.

**Abstract:**

Prostate cancer (PCa) is the second most frequent type of cancer in men worldwide, with 288,300 new cases and 34,700 deaths estimated in the United States in 2023. Treatment options for early-stage disease include external beam radiation therapy, brachytherapy, radical prostatectomy, active surveillance, or a combination of these. In advanced cases, androgen-deprivation therapy (ADT) is considered the first-line therapy; however, PCa in most patients eventually progresses to castration-resistant prostate cancer (CRPC) despite ADT. Nonetheless, the transition from androgen-dependent to androgen-independent tumors is not yet fully understood. The physiological processes of epithelial-to-non-epithelial (“mesenchymal”) transition (EMT) and mesenchymal-to-epithelial transition (MET) are essential for normal embryonic development; however, they have also been linked to higher tumor grade, metastatic progression, and treatment resistance. Due to this association, EMT and MET have been identified as important targets for novel cancer therapies, including CRPC. Here, we discuss the transcriptional factors and signaling pathways involved in EMT, in addition to the diagnostic and prognostic biomarkers that have been identified in these processes. We also tackle the various studies that have been conducted from bench to bedside and the current landscape of EMT-targeted therapies.

## 1. Introduction

Prostate cancer (PCa) is the second most frequent type of cancer in men worldwide, with 288,300 new cases and 34,700 deaths estimated in the United States in 2023 [1,2,3]. Mortality rates for PCa have decreased in recent years with the use of prostate-specific antigen (PSA) testing; however, there remains a significant disease burden [4,5]. The most common type of PCa is adenocarcinoma, which is graded using the Gleason scoring system [2]. This system is based on the architectural growth patterns of the tumor, with Gleason pattern 1 showing discrete, well-formed small round glands and Gleason pattern 5 showing sheets of tumor cells or individual cells with no gland formation [6,7]. The International Society of Urologic Pathology (ISUP) has recently introduced the Grade Group system, which provides more accurate stratification than the Gleason score alone [8].

Treatment options for early-stage PCa include external beam radiation therapy, brachytherapy, radical prostatectomy, active surveillance, or a combination of these [2]. In more advanced cases, androgen-deprivation therapy (ADT) is considered the first-line therapy, given that PCa cells rely on the androgen receptor (AR) for growth and survival [2,9,10]. Despite these interventions, PCa in many patients eventually progresses to castration-resistant prostate cancer (CRPC), marking the transition from an androgen-dependent to an androgen-independent state. This process is not fully understood; however, continuous AR signaling, *AR* gene amplification, mutations, ligand-independent activation, coregulators, and cancer stem cell (CSC) recruitment are thought to be involved [9,11,12,13,14,15,16,17].

## 2. EMT and MET as Targets for Therapy

In general, epithelial cells exhibit a tight-junction, tightly packed morphology, while non-epithelial (“mesenchymal”) cells are characterized by loose packing and increased motility [18,19]. The physiological processes of epithelial-to-non-epithelial (“mesenchymal”) transition (EMT) and mesenchymal–epithelial transition (MET) play essential roles in normal embryonic development [20]. Early on, EMT has been linked to the initial generation of the three germ layers from pluripotent stem cells [21]. Subsequently, EMT leads epithelial cells to acquire non-epithelial (mesenchymal) characteristics, as noted by the loss of E-cadherin and the gain of vimentin and N-cadherin [21,22,23]. This enables cells to disengage from tight junctions and gain mobility to migrate to other tissues [19]. MET complements this process by orchestrating the formation of organized structures once the EMT-induced cells have arrived at their proper location [21]. Beyond embryology, EMT has been shown to also be involved in the migratory processes implicated in wound healing, tissue regeneration, and organ fibrosis [19].

**Figure 1:**
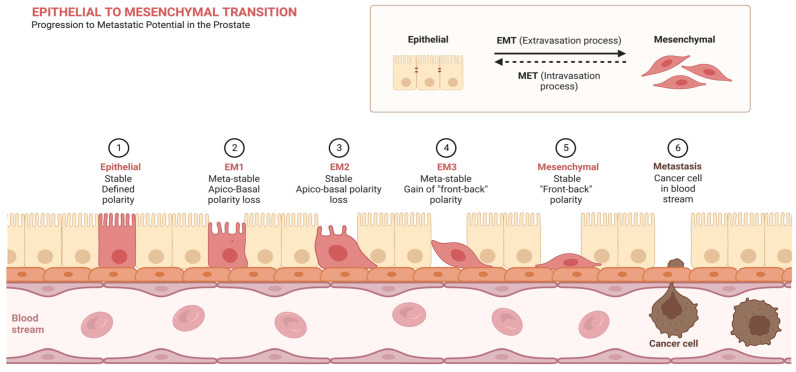
Mechanism of action of EMT in the prostate cell. The progression to metastatic cancer involves the loss of stable apico-basal epithelial cell polarity, the gain of front–back polarity, and eventual metastasis to the bloodstream. Created with BioRender.com (accessed on 11 March 2023).

In malignancy, the processes of EMT and MET are often dysregulated to promote cancer progression. Each has been linked to the induction of CSCs capable of generating new tissue [17,21,24], as well as the promotion of enhanced mobility, tissue invasion, and therapy resistance [25]. Because of these factors, EMT and MET have been identified as important targets for novel cancer therapies, including CRPC (Figure 1).

## 3. Epithelial-To-Mesenchymal Transition in Cancer Progression

### 3.1. EMT Initiation

EMT in cancer cells has been implicated in tumor initiation, malignant transformation, CSC survival, metastasis, and treatment resistance [26]. These associations are primarily due to the sequential loss of epithelial characteristics, such as E-cadherin expression that maintains cell–cell interactions, in favor of a non-epithelial (“mesenchymal”) phenotype capable of tissue invasion. The initiation of EMT involves a variety of factors intrinsic to the tumor microenvironment (TME), including growth factors and cytokines, hypoxia, and interactions with the extracellular matrix (ECM) [17].

Some of the best-characterized growth factors and cytokines involved in EMT induction include transforming growth factor beta (TGF-β), hepatocyte growth factor (HGF), fibroblast growth factor (FGF), epidermal growth factor (EGF), and platelet-derived growth factor (PDGF). TGF-β is the most studied of these factors, with a variety of tumor mutations leading to augmented expression and EMT activation, primarily through *SMAD*-mediated signaling [27]. Elevated HGF expression has been observed in cancer-associated fibroblasts of the colon, breast, pancreas, and prostate [28]. Enhanced expression of FGF has been connected to an increase in the expression of non-epithelial markers, such as vimentin and FSP-1, as well as the promotion of metalloproteases (MMPs) and cytoskeletal rearrangements [29,30]. EGF overexpression plays a prominent role in various cancers including the breast, prostate, cervix, and head and neck via PI3K/Akt signaling [31]. Excessive PDGF expression has been observed in cancers of the prostate, lung, kidney, ovary, brain, and pancreas and is involved with multiple important EMT pathways, including PI3K/Akt, Notch, and others [32].

Hypoxia commonly occurs in the microenvironment of solid tumors due to overcrowding and impaired diffusion, leading to the inhibition of prolyl hydroxylases and a resultant upregulation of hypoxia-inducible factors (HIFs). With regards to EMT induction, HIF-1-alpha is of particular interest and has been associated with TGF-β signaling, as well as the SMAD, Ras/MEK/ERK, and PI3K/Akt signaling pathways [33]. This hypoxia-driven mechanism has been observed in many different cancers, including breast, ovarian, lung, prostate, and pancreatic cancers, among others [33].

The ECM maintains proper tissue segregation and is a major regulator of intracellular signaling cascades. As such, cancer progression relies heavily on manipulating the ECM, a role for which EMT is particularly well suited. EMT utilizes a dynamic composition of integrins to connect with certain aspects of the ECM, such as type I collagen, which is well known to be augmented in a variety of cancers [34]. The interaction with a2b1 integrin has been investigated and correlated with EMT induction in breast, lung, and pancreatic cancers via NF-kB, JNK, and TGF-β pathway activation [35,36,37,38]. Additionally, this action has been observed to directly suppress E-cadherin and indirectly induce N-cadherin, an important early transition in EMT [38]. As EMT progresses, the transitioning cell expands secretion of type 1 collagen and fibronectin, enhancing integrin activation and producing a network by which the transitioning cell can utilize lamellipodia and filopodia for migration [39]. This enhanced mobility is complemented by the induction of various MMPs that cleave type IV collagen in the basal lamina and disrupt epithelial cell junctions, both of which facilitate cell invasion [40]. Furthermore, MMPs have demonstrated an ability to directly induce EMT progression, as evidenced by MMP-3 enhancement of the transcription factor SNAI1 in lung cancer [41].

### 3.2. EMT Transcription Factors

The cellular alterations seen with EMT result from changes in gene expression that are primarily driven by EMT-regulating transcription factors (EMT-TFs). EMT-TFs are themselves induced through a variety of direct and indirect signaling pathways upregulated in cancer cells. A large number of EMT-TFs have been identified; however, the most well-studied families are SNAIL, TWIST, and zinc-finger E-box-binding homeobox (ZEB) [42].

The SNAIL family of EMT-TFs includes SNAI1 (Snail), SNAI2 (Slug), and SNAI3 (Smuc); however, SNAI3 is a poor EMT-inducer [43,44]. They are most notable for accumulating in the cell nucleus and binding the *CDH1* promoter to suppress the transcription of its encoded protein, E-cadherin [45]. SNAIL expression is regulated through multiple signaling pathways, including receptor tyrosine kinases (RTKs), TGF-β, Notch, Wnt, and others, as well as post-translational modifications [44]. Enhanced SNAIL expression has been correlated with a higher tumor grade and metastatic potential in breast, ovarian, and hepatocellular carcinomas [46,47,48]. Furthermore, this propensity for EMT-induced metastasis has been shown to involve immunosuppression, with SNAIL-knockdown leading to inhibited tumor growth and metastasis from significant elevations in tumor-infiltrating and systemic immune responses [49].

The TWIST family of EMT-TFs includes TWIST1 and TWIST2. TWIST1 has been shown to directly bind the E-cadherin promoter to repress its expression as well as bind the E-box cis-element in the N-cadherin gene to enhance its expression [50,51]. Additionally, TWIST1 can bind the SNAI2 promoter to enhance SNAIL-mediated EMT induction [52]. TWIST expression is regulated through multiple signaling pathways, including RTKs, TGF-β, Notch, Wnt, TNF-alpha, HIF-1-alpha, and others, as well as post-translational modifications [53]. Enhanced TWIST expression has been observed in many cancers, including breast, bladder, gastric, hepatocellular carcinoma, and others, and is associated with higher tumor grade, metastasis, and therapeutic resistance [50].

The ZEB family of EMT-TFs includes ZEB1 and ZEB2. Both have been shown to bind E-box regions around the *CDH1* promoter to suppress the expression of E-cadherin [54]. ZEB1 has also been shown to repress transcription of the epithelial cell polarity genes *HUGL2, Crumbs3*, and *PATJ* [55]. ZEB expression is regulated through multiple signaling pathways, including RTKs, TGF-β, Notch, Wnt, and others, as well as post-translational modifications [56]. Enhanced ZEB expression has been observed in many cancers, including prostate, bladder, brain, breast, cervical, colon, and others, and is associated with a higher tumor grade, metastasis, and therapeutic resistance [56].

### 3.3. EMT Signaling Pathways

#### 3.3.1. TGF-β Signaling

TGF-β signaling is a well-known pathway of EMT induction. Its ligands include isoforms from either the TGF-β or bone morphogenic protein (BMP) families, with TGF-β-1, BMP2, and BMP4 being particularly associated with EMT [57,58,59]. Ligand initially binds to one of two types of serine and threonine kinase receptors, type I receptors, of which there are seven, or type II receptors, of which there are five [60]. Binding leads to the formation of a TGF-β heterotetrameric receptor complex with type II receptors trans-phosphorylating the type I receptors, activating their kinase activity [60]. This induces various signaling cascades, most notably involving SMAD2 and SMAD3, which oligomerize with SMAD4 for nuclear localization [60]. Inside the nucleus, the SMAD complex binds regulatory elements that induce the expression of various EMT genes, including SNAI1/2, TWIST1/2, and ZEB1/2 [61]. TGF-β signaling can also utilize SMAD-independent pathways, including PI3K/Akt and Ras/MEK/ERK [60]. Activated Akt2 has been shown to enhance the translation of the EMT-inducers DAB2 and ILE1, as well as SNAI1 [62,63,64]. Additionally, MEK/ERK stimulation plays a demonstrated role in the delocalization of zonula occludens and E-cadherin from epithelial cell junctions, driving EMT progression [65].

#### 3.3.2. Receptor Tyrosine Kinase Signaling

Receptor tyrosine kinase (RTK) signaling includes a broad range of pathways initiated by a variety of ligands implicated in EMT, including HGF, FGF, PDGF, EGF, and insulin-like growth factor (IGF). In short, ligand binding to RTKs leads to receptor dimerization and trans-phosphorylation of intracellular domains, initiating additional signaling cascades via Ras, PI3K, FAK, Src, and TAK [66]. Of note, there is a good amount of overlap with the SMAD-independent pathways described with TGF-β signaling, particularly with regards to the Ras/MEK/ERK and PI3K/Akt cascades [67]. Beyond what was previously described, IGF-1 and HGF have been shown to utilize ERK signaling to enhance ZEB1 in PCa cells and SNAI1/2 expression in hepatocellular carcinoma cells, respectively [68,69]. PDGF operates through the PI3K/Akt pathway to augment the transcription of *CDH2*, leading to increases in N-cadherin [70]. *FAK* activation plays an essential role in upregulating *SNAIL* and *TWIST* transcription as well as downregulating E-cadherin expression and promoting its internalization [71]. Increased Src expression has been demonstrated in CRPC and correlated with a greater prevalence of distant metastases [72,73]. Src-suppression in breast carcinoma cells demonstrated an increase in E-cadherin and a decrease in vimentin, reducing the apparent metastatic potential [74]. Similarly, elevations in Src expression showed downregulation of E-cadherin with an apparent increase in pancreatic ductal carcinoma invasiveness [75].

#### 3.3.3. Wnt Signaling

Wnt signaling is activated by the binding of the Wnt ligand to a Frizzled receptor and a lipoprotein receptor-related protein (LRP) [76]. This allows GSK-3-beta to phosphorylate LRP, which recruits Dishevelled and Axin to the plasma membrane, allowing beta-catenin to translocate to the nucleus [76]. Intranuclear beta-catenin is able to form a complex with LEF-1 that leads to the inhibition of *CDH1* transcription, suppressing E-cadherin production [77]. Beta-catenin has also been shown to directly induce SNAI1 and SNAI2 expression in a number of cancers, as well as promote TWIST expression in mammary epithelial cells [78,79,80].

#### 3.3.4. Notch Signaling

Notch signaling involves an intercellular interaction between the extracellular domain of the Notch receptor and its cell surface ligands, Delta and Jagged [81]. This stimulates proteolytic cleavage of the Notch receptor intracellular domain by ADAM-MMPs and gamma-secretase, allowing for translocation to the nucleus [81]. Once in the nucleus, the cleaved Notch receptor is able to activate the expression of various genes implicated in EMT, including NF-kB, Akt, and p21, as well as directly promote SNAIL expression [82,83,84]. Furthermore, Notch signaling is capable of inducing HIF-1-alpha release, leading to the upregulation of LOX and subsequent stabilization of SNAI1 [85]. Furthermore, the inhibition of Notch signaling in lung adenocarcinoma has demonstrated reductions in tumor invasiveness and EMT progression [86].

#### 3.3.5. Hedgehog Signaling

Hedgehog (Hh) signaling is mediated through the binding of Hh ligands to PTCH receptors, resulting in the internalization and degradation of the Hh-PTCH complex [87]. This releases the inhibition on Smoothened, which initiates an intracellular cascade that activates the Gli family of transcription factors [87]. Gli1 has been shown to promote the transcription of SNAI1 and has been implicated in promoting EMT-mediated invasion of ileal neuroendocrine tumors [88,89]. Hh signaling is notable for being highly involved in crosstalk with other pathways that promote EMT. Hh induction of TGF-β-1 signaling has been correlated with increased motility and invasiveness in gastric cancer [90]. Hh enhancement of JAG2 expression has been shown to augment the Notch pathway [91]. Furthermore, the inhibition of Hh signaling in pancreatic cancer cells has demonstrated the impairment of EMT-mediated disease progression [92].

## 4. Studies on EMT in Prostate Cancer

Different studies have elaborated on the role of androgens, signaling pathways, epigenetic alterations, TME, and CSCs in PCa pathogenesis. First, androgens play a critical role in the development of PCa. Specifically, AR is considered a key mediator of PCa growth, including the induction of cell cycle progression, inhibition of apoptosis, and activation of angiogenesis. Second, various signaling pathways are implicated in PCa development and progression. Those include the TGF-β, RTK, WNT, Notch, and Hedgehog signaling pathways. These pathways promote PCa growth by inducing EMT and promoting PCa stemness properties. Third, epigenetic and TME alterations also contribute to PCa development and progression.

### 4.1. In Vitro and In Vivo Studies

#### 4.1.1. EMT Surface Markers

Vimentin is an important cell surface marker of mesenchymal cells and, as such, can be utilized to identify the occurrence of EMT. Studies have found increased co-expression of cytokeratin 8 and vimentin in androgen-independent CRP [16,93]. A follow-up study conducted by the same group sought to determine whether an increase in these biomarkers was associated with worse clinical outcomes [94]. A total of 122 samples from patients with PCa were evaluated, and it was found that these markers were associated with a higher Gleason score. This study also confirmed that the degree of EMT progression is predictive of PSA failure regardless of the Gleason score, pathological state, or surgical margins [94].

#### 4.1.2. EMT Transcription Markers

Evidence indicates that tumors may originate from CSCs that express ZEB1 in prostatic basal stem cells, triggering the induction of EMT with stem cell traits, immune evasion, and epigenomic reprogramming [95]. Basal cells exhibit intrinsic stem-like and neurogenic properties, characterized by genes that are enriched in advanced, anaplastic, castration-resistant, and metastatic PCa [95]. Single-cell RNA-sequencing analysis holds promise for uncovering detailed transcriptomic signatures that can help uncover the lineage contribution to CSCs and their association with PCa progression, drug resistance, and metastasis [95].

In metastatic CRPC (mCRPC), epigenetic reprogramming, especially through polycomb repression, is thought to underlie lineage plasticity [96]. The polycomb repressive complex plays a crucial role in regulating EMT, with Hsp90 acting through EZH2 to reverse its function, leading to tumor growth and tissue invasion [96]. EZH2 also promotes neuroendocrine differentiation through histone methylation at H3 lysine 27, with this differentiation being a significant marker of certain PCa cell lines [96]. Furthermore, polycomb regulation modulates stem cell functions.

In a study utilizing Pten knockout mice, Rb1 loss was found to be a significant driver of lineage plasticity in a Pten loss-induced prostate adenocarcinoma model, as evidenced by an increase in EMT and stemness [97]. Transcriptomic profiling revealed that this phenotype was mediated by *SOX2* and *EZH2*, both of which are epigenetic reprogramming factors. Additional studies have demonstrated a strong link between *TP53*, *RB1*, lineage plasticity, and epigenetic changes that contribute to CRPC [97].

EMT represents a mechanism by which cancer cells can acquire resistance to therapy, including resistance to chemotherapy, radiotherapy, increased drug efflux, and evasion of apoptosis [98]. Importantly, Snail has been shown to prevent treatment-induced apoptosis by interfering with Tp53 or Pten [98]. Two studies utilizing mouse models demonstrated that primary and secondary tumor cells gain therapy resistance through an EMT-dependent mechanism [93,98].

#### 4.1.3. Tumor Microenvironment

The crosstalk between epithelial tumor cells of PCa and surrounding stroma within the tumor microenvironment plays a crucial role in the progression of the disease into its advanced stages and eventual metastasis. Some of the key players within the stroma include mesenchymal stem/progenitor cells, stromal-derived mediators of inflammation, regulators of angiogenesis, connective tissue growth factors, wingless homologs (Wnts), and integrins [17]. A study by Zhou et al. referred to the mechanism of neuroendocrine differentiation that occurs in parallel with castration resistance development in advanced PCa [99]. In addition, it is noteworthy mentioning that the TME evolves in parallel with the PCa clones, where the ECM and vasculature architecture is altered, recruiting specialized tumor-supporting cells that favor tumor spread and colonization at distant sites, particularly the bones where a premetastatic niche is orchestrated [100].

An early study investigating the creation of the TME in PCa highlighted the essential role of cancer-associated fibroblasts (CAFs) [101]. CAFs were found to acquire stemness and an EMT phenotype after interacting with cancer-induced macrophages in the TME. This induction event was subsequently correlated with increased PCa metastasis [101].

A study by Bezzi and colleagues showed that different genetically engineered mouse models with homozygous Pten loss exhibited varied immune compositions in the TME [102]. They demonstrated that the loss of Zbtb7a along with Pten resulted in higher CXCL5 expression, while the loss of Tp53 along with Pten led to increased CLCL17 expression, potentially attracting myeloid cells to the TME through distinct mechanisms [102].

A study conducted by Su and colleagues demonstrated that PRC1 drives the metastasis of certain PCa subtypes through the regulation of CCL2 expression [103]. In the TME, CCL2 was shown to promote PCa self-renewal, angiogenesis, and immune system suppression. They also found that PRC1 combined with immune checkpoint blockade effectively suppresses metastasis in PCa-induced mice, suggesting that CCL2 expression could serve as a biomarker for response to therapy [103]. A separate study found that the inactivation of AR reduces a transcriptional repressor of CCL2, which mediates EMT of prostate tumor cells [93].

#### 4.1.4. TGF-β Signaling

TGF-β is a well-known EMT-inducer via alternative splicing of CD44, forming an isoform capable of migration, invasion, and tumor initiation. An in vitro study using human PCa cells treated with TGF-β showed a decrease in E-cadherin and an increase in N-cadherin, among other EMT and CSC markers [104]. The same study also performed in vitro and in vivo investigations that showed that CD44 promoted PCa cell migration, invasion, and tumor initiation [104].

#### 4.1.5. RTK Signaling

Through both in vivo and in vitro studies, Bluemn and colleagues evaluated the inevitable shift to CRPC through androgen independence [105]. While they observed FGF to be markedly overexpressed in CRPC, the expression of the FGF receptor (FGFR), an RTK, was also found to have increased expression [105,106]. The enhanced activity of these specific RTKs was associated with ligand-independent activation of AR transcription in these models [106]. Another study that used xenograft growth demonstrated that PCa expression of IL6 is capable of activating a specific type of CAF that induces EMT invasiveness and stemness [107]. These findings suggest IL6 and FGF as potential biomarkers.

#### 4.1.6. WNT Signaling

A study performed by Acevedo and colleagues identified FGF-receptor-mediated EMT in PCa progression that utilized SOX9 and Wnt signaling [108]. It was found that, in inducible FGFR1 prostate mouse models, activation with chemical inducers of dimerization led to highly synchronous, stepwise progression to adenocarcinoma linked to EMT [108].

A previous study demonstrated that the loss of *PTEN* can initiate EMT [109]. Another study found that restoring *PTEN* in breast cancer prevented EMT and stemness through the downregulation of Abelson interactor 1 (*Abi1*) [110]. Abi1 is an adapter protein that uses Wnt signaling to regulate the progression of epithelial plasticity in PCa [109].

#### 4.1.7. Notch Signaling

One study has implicated the Notch pathway as an EMT promoter in PCa, with recent research describing it as an important player through activation of the estrogen receptor through the use of castrated mice [111]. The estrogen receptor alpha is expressed in the basal cell layers of the normal prostate and has key roles in coordinating stem cells for prostate development [111]. It was also found that EZH2 was recruited by estrogen receptors and facilitated the binding of these receptors to the Notch promoter [111].

#### 4.1.8. Hedgehog Signaling

A study performed by Ishii and colleagues determined that the Shh-inhibitor vismodegib prevented EMT in CRPC cells, resulting in decreased tumor growth in mice when compared to controls. It was also shown to inhibit cancer cell proliferation via enhanced apoptosis [112].

#### 4.1.9. PI3K/AKT Signaling

Contactin1 (Cntn-1) is an immunoglobulin superfamily cell adhesion neuronal membrane glycoprotein that promotes metastasis through EMT [113]. Cntn-1 downregulation has been shown to decrease PI3K/Akt signaling activity, an important pathway for EMT propagation [114]. This signaling pathway promotes EMT in PCa through the upregulation of EMT-inducing transcription factors and the activation of downstream effectors, such as mTOR and GSK-3β [115]. Activation of the PI3K/AKT pathway upregulates EMT-inducing transcription factors, such as *Snail*, *Slug*, and *ZEB1*, which in turn repress the expression of epithelial markers, such as E-cadherin, and induce the expression of mesenchymal markers, such as N-cadherin, vimentin, and fibronectin [94]. This results in a loss of cell–cell adhesion and an increase in cell motility and invasiveness.

Normal prostatic epithelium in rodents is composed of basal, secretory luminal, and neuroendocrine cells, with stem cells identified in both basal and luminal lineages [116]. One study suggested that PCa tumors may arise from CSCs with Pten/Akt signaling dysregulation, resulting in a heterogenous population of cells similar to those present in the normal prostatic epithelium [116].

#### 4.1.10. Integration of the Different Signaling Pathways in PCa Progression

The various signaling pathways mentioned above collectively play a role in the progression of PCa. The TGF-β pathway induces EMT via alternative splicing of *CD44*, leading to increased migration, invasion, and tumor initiation. Likewise, *RTK* signaling, specifically *FGF* and *FGFR*, is overexpressed in CRPC, leading to ligand-independent activation of *AR* transcription and increased invasiveness and stemness. The *WNT* signaling pathway is also implicated in EMT, where the loss of *PTEN* initiates this process and promotes stemness through the regulation of *Abi1*. The Notch pathway also promotes EMT, with estrogen receptors and *EZH2* recruiting to the Notch promoter to activate EMT. The interplay between the different pathways highlights their involvement in PCa progression.

### 4.2. Translational and Clinical Studies

Transcriptional analysis has identified distinct gene signatures associated with various EMT intermediate states, which have facilitated the identification of EMT transcriptional promoter genes that could serve as biomarkers [117]. The analysis of PCa has revealed that epithelial plasticity is directly correlated with poor clinical prognosis [118]. Notably, recent research by Stylianou and colleagues found that EMT biomarkers were enriched in PCa patients who had undergone ADT, which selects for wide-scale transcriptional changes in ADT-resistant tumor cells [118]. *SNAI1* was found to be the primary driver of EMT in their PCa model, with subsequent targeting of *SNAIL* leading to reduced mesenchymal drivers, such as ZEB1, and the re-expression of epithelial markers, such as E-cadherin [119]. Additionally, the loss of *SNAI2* led to a better response to ADT [119]. It was also found that the Wnt pathway Wnt5a/Fzd2 was found to increase EMT markers and predict PCa aggressiveness, while Abi1 controlled epithelial plasticity downstream of the Wnt receptor Fzd2 [109].

A study by Jedroszka and colleagues divided patients into groups based on their expression levels of AR, ESR1, and ESR2 [120]. It was found that in those under the age of 50, there was a completely different expression of EMT genes than in those over the age of 50 [120]. After the investigation of 43 genes involved in EMT, it was found that those under 50 overexpressed *CTNNB1*, *CDH1*, *SMAD2*, *SMAD3*, *TCF3*, and *LEF1*, while those over 50 overexpressed Snail1 and underexpressed *KRT5*, *KRT19*, *OCLN*, *CDH2*, and *MUC1* [120]. The difference in gene expression also predicted the presence of a more aggressive, invasive phenotype in those under 50, regardless of the Gleason score [120]. The reasons for the change in gene expression observed are not fully understood. However, it has been suggested that age-related changes in the hormonal milieu, especially the decrease in androgen levels, may contribute to this phenomenon. Additionally, aging is associated with various epigenetic changes, including alterations in DNA methylation, histone modifications, and non-coding RNA expression, which can impact gene expression. Further research is needed to fully elucidate the mechanisms underlying the age-related differences in EMT gene expression in PCa.

In a study by He and colleagues, single-cell analysis of advanced PCa patients treated with ADT revealed the co-expression of multiple AR isoforms, with resistance to therapy associated with upregulation of EMT and TGF-β gene signatures [121]. The study also found a subset of patient tumors with high expression of dysfunctional cytotoxic CD8+ T cell markers, indicating a potential impact of EMT on immune responses in CRPC [121].

## 5. Potential Biomarkers of Interest for Targeted Therapy

Based on the known pathways involved in EMT and prior in vitro, in vivo, translational, and clinical studies, many biomarkers have been identified as potential targets for EMT-targeted therapy in PCa. For EMT-driver targeting, the genes for TGF-β, PRC1/2, SNAI1/2, FGF, CNTN1, and BRD4, as well as the transcription factors SOX2, EZH2, and HSP90, have been proposed. For EMT-effector targeting, the genes for ZEB1/2, TWIST1/2, EZH2, Kaiso, ABI1, and CDH1/2, as well as the transcription factors ZEB1, LSD1, and PRC1/2, have been proposed. Due to the crucial role each of these factors plays in regulating the EMT process, inhibiting their expression may be a way of reversing the EMT process and preventing the activation of these pathways. For EMT-stemness targeting, the genes for SOX2 and PRC1/2 have been proposed. The immune targets of IL6, CCL2, and CXCL5 have also been identified as potential targets. Two important biomarkers involved in the cell cycle that have been identified are Tp53 and Rb1. Furthermore, many of these identified biomarkers play roles in various EMT signaling pathways, including RTK, TGF-β, NF-kB, Wnt, PI3K/Akt, PPAR, and Notch. Therefore, inhibitors of these pathways may also have investigational importance. Levels of E-cadherin, vimentin, and N-cadherin may also be useful markers of EMT and MET conversion.

## 6. Clinical Trials

A number of clinical trials investigating the aforementioned biomarkers were listed on https://clinicaltrials.gov/ (accessed on 15 February 2023). For the purposes of this review, only those clinical trials with specific targeted therapies for the listed biomarkers or those using vimentin and/or N-cadherin as a marker of decreased EMT were included for further analysis, as seen in Table 1.

**Figure 2:**
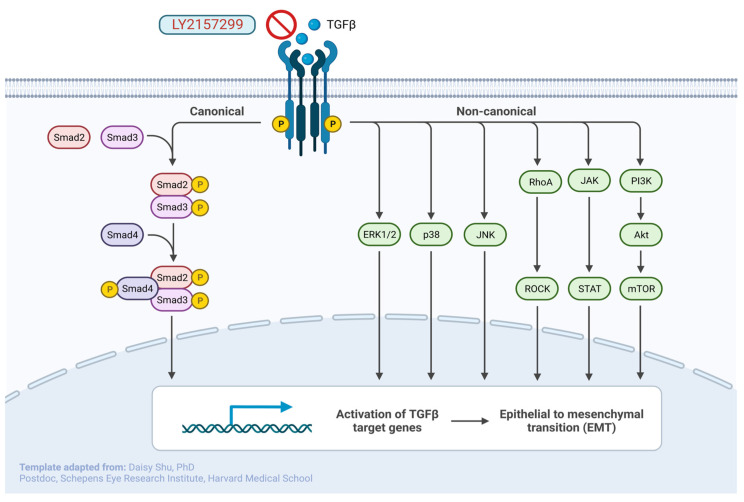
Mechanism of action of the TGF-β inhibitor LY2157299. As shown, inhibition of TGF-β causes downstream inhibition effects on SMAD, ERK1/2, p38, JNK, RhoA/ROCK, JAK/STAT, and PI3K/Akt/mTOR, leading to decreased activation of TGF-β target genes, and a decrease in EMT. Created with BioRender.com (accessed on 11 March 2023).

**Figure 3:**
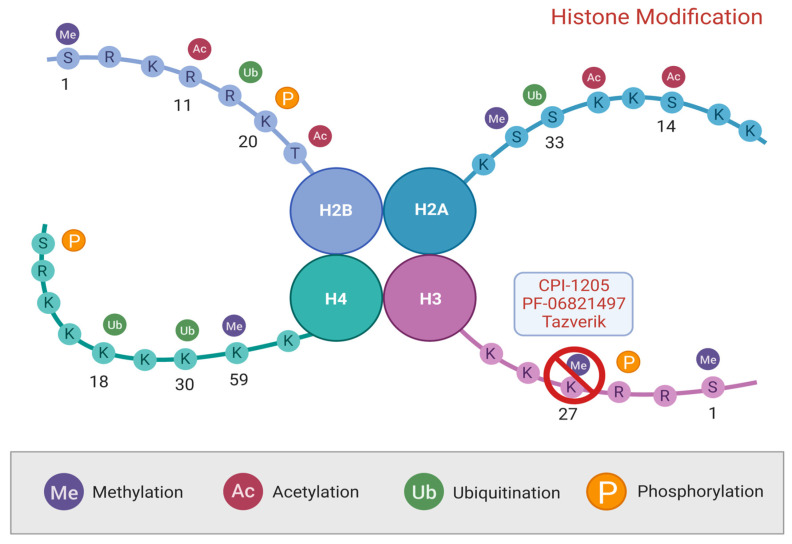
Mechanism of action of the EZH2 inhibitors CPI-1205, PF-06821497, and Tazverik. EZH2 is an enzyme responsible for methylation of histone H3 at lysine 27. Created with BioRender.com (accessed on 11 March 2023).

Analysis of these trials revealed many investigational therapies of interest. Clinical trial *NCT02452008* is recruiting for a study involving LY2157299, a TGF-β receptor inhibitor. Clinical trial *NCT05413421* is recruiting for a study involving ORIC-944, a highly selective, allosteric, small-molecule inhibitor of PRC2 (Figure 2). Dovitinib, an RTK inhibitor with unique inhibitory effects on FGF, underwent investigation in clinical trials *NCT01741116*, *NCT01994590*, and *NCT02065323*. Various EZH2 inhibitors, including CPI-1205, PF-06821497, and Tazverik, are being studied in clinical trials *NCT03480646*, *NCT03460977*, *NCT05567679*, and *NCT04179864*, all of which are currently recruiting. Completed studies involving HSP90 inhibitors AT13387 and STA-9090 were conducted in clinical trials *NCT01685268* and *NCT01270880*. *NCT02140996*, which investigated the Ad-sig-hMUC-1/ecdCD40L vector vaccine meant to disrupt E-cadherin, currently has an unknown status and was last updated in 2016. JBI-802, an LSD1/HDAC6 inhibitor, is being studied in clinical trial *NCT05268666*, which is currently recruiting. *NCT00433446*, *NCT00385827*, and *NCT00401765* have finished studies involving CNTO-328, an anti-IL6 chimeric monoclonal antibody. *NCT00992186* has been completed and involved Carlumab, an anti-CCL2 therapy (Figure 3).

Of the twenty clinical trials included, seven have been completed, with only three of those having available results. In clinical trial *NCT01270880*, the progression-free survival of individuals with mCRPC who had previously received docetaxel and were treated with STA-9090 was investigated. At the conclusion of phase II, all participants experienced disease progression. In clinical trial *NCT00433446*, the proportion of participants with a PSA response to CNTO-328 was studied, and it was found that only 3.8% of participants experienced a decrease in PSA of at least 50% from baseline. Finally, clinical trial *NCT00992186* evaluated the safety and efficacy of Carlumab, and it was determined that none of the participants experienced any form of composite response at the end of the trial.

There are currently no clinical trials listed that involve therapies specifically targeting PRC1, SNAI1/2, CNTN1, BRD4, SOX2, ZEB1/2, TWIST1/2, Kaiso, ABI1, CXCL5, Tp53, or Rb1.

## 7. Conclusions and Future Directions

The association of EMT with the progression of cancer grading, metastasis, and therapeutic resistance has been of great investigational importance in recent years. Identifying relevant biomarkers for EMT is essential for the development of therapeutic interventions, particularly in heavily treatment-resistant diseases such as CRPC, where EMT promotes invasion and CSC survival. While there are currently many known biomarkers for EMT, more are being identified with the help of transcriptional analysis. The majority of the analyses performed on the clinical utility of these targets have been through in vivo or in vitro studies, with few being conducted through translational applications.

Of the targets identified in this review, only a small minority of those found on clinicaltrials.gov included investigational therapies. While there were twenty clinical trials found on TGF-β, PRC2, FGF, EZH2, HSP90, CDH1, LSD1, IL6, CCL2, N-cadherin, and vimentin, only seven have been completed at this time, with three of these having posted results. To date, the outcomes of these completed trials do not support EMT biomarker-targeted therapy as an effective means of treatment for PCa, which is surprising given the strength and breadth of investigations assessing EMT involvement in cancer progression. The trial examining STA-9090, an HSP90 inhibitor, showed that all participants had disease progression. The trial involving CNTO-328, an anti-IL6 chimeric monoclonal antibody, concluded that only 3.8% of participants experienced a significant decrease in PSA. Lastly, the trial looking at Carlumab, an anti-CCL2 therapy, indicated that none of the participants experienced a significant response to treatment. Given these disappointing outcomes, the results of the remaining 17 clinical trials are of great importance in guiding the future application of EMT biomarker therapy in clinical practice. Additionally, the results of clinical trials comprising targeted therapies of PRC1, SNAI1/2, CNTN1, BRD4, Sox2, ZEB1/2, TWIST1/2, Kaiso, ABI1, CXCL5, TP53, and RB1 are increasingly important. Finally, since none of the studies found on clinicaltrials.gov implicated the specific application of these therapies for the investigation of EMT, future studies involving these factors should be expected.

## Figures and Tables

**Table 1:** List of clinical trials involving the EMT biomarkers of interest with investigational therapies targeting those biomarkers (https://clinicaltrials.gov/, accessed on 15 February 2023).

Target	NCT Number	Phase	Status	Outcome of Interest
TGF-β	*NCT02452008*	2	Recruiting	Compare the progression-free survival of those with mCRPC who are treated with enzalutamide alone vs. enzalutamide plus LY2157299, a TGF-β receptor inhibitor.
PRC2	*NCT05413421*	1	Recruiting	Establish a phase 2 dose and/or MTD of ORIC-944, a potent, highly selective, allosteric, orally bioavailable, small molecule inhibitor of PRC2.
FGF	*NCT01741116*	2	Completed	Evaluate the efficacy and safety of Dovitinib, an RTK inhibitor with unique inhibitory effects on FGF. No results posted.
	*NCT01994590*	2	Terminated	Evaluate the safety of adding Dovitinib to abiraterone acetate and prednisone in those with mCRPC. Terminated due to sponsor no longer supplying study drug.
	*NCT02065323*	2	Withdrawn	Evaluate if adding Dovitinib to ADT will prolong time to disease progression in those with mCRPC receiving ADT for the first time. Withdrawn due to budgeting considerations and time length to development.
EZH2	*NCT03480646*	1, 2	Active, not recruiting	Determine the dose-limiting toxicities in those with mCRPC receiving CPI-1205, a small molecule inhibitor of EZH2.
	*NCT03460977*	1	Recruiting	Evaluate the safety and efficacy of PF-06821497, an EZH2 inhibitor, in those with CRPC.
	*NCT05567679*	1	Not yet recruiting	Evaluate if the underlying prostate cancer tumor is more sensitive to the patient’s immune system after receiving radical prostatectomy following preoperative treatment with Tazverik, an EZH2 inhibitor.
	*NCT04179864*	1, 2	Recruiting	Determine the safety and efficacy of combining Tazverik with either enzalutamide or abiraterone/prednisone in those with CRPC who have not received chemotherapy.
HSP90	*NCT01685268*	1, 2	Completed	Determine the safety and antitumor activity of AT13387, an HSP90 inhibitor, either alone or in combination with abiraterone. No results posted.
	*NCT01270880*	2	Completed	Evaluate the progression-free survival of those receiving STA-9090, an HSP90 inhibitor, with mCRPC refractory to docetaxel. At 6 months, all 18 participants had disease progression.
CDH1	*NCT02140996*	1	Unknown	Assessment of safety and dose level of Ad-sig-hMUC-1/ecdCD40L vector vaccine meant to disrupt e-cadherin. Last status update in 2016 listed it as recruiting.
LSD1	*NCT05268666*	1, 2	Recruiting	Assessment of the MTD and efficacy of JBI-802, an LSD1/HDAC6 inhibitor.
IL6	*NCT00433446*	2	Completed	Determine PSA response to CNTO-328, an anti-IL6 chimeric monoclonal antibody, in those with mCRPC. 3.8% of participants had a reduction in PSA of at least 50%.
	*NCT00385827*	2	Terminated	Determine the number of participants with adverse events and the progression-free survival in those receiving CNTO-328 for mCRPC. Terminated after determination of a lack of efficacy.
	*NCT00401765*	1	Completed	Determine the safety and efficacy of CNTO-328 in combination with docetaxel in those with mCRPC. No results posted.
CCL2	*NCT00992186*	2	Completed	Determine the safety and efficacy of Carlumab, an anti-CCL2 therapy, in those with mCRPC. Of 41 participants, 0 had a composite response as measured by: a change in skeletal lesions, extra-skeletal lesions, or PSA.
N-Cadherin and Vimentin	*NCT01990196*	2	Active, not recruiting	Measure vimentin and N-cadherin expression following radical prostatectomy and treatment with degarelix, enzalutamide, trametinib, or dasatinib, which are capable of SRC and/or MEK inhibition of tyrosine kinase.
	*NCT02204943*	2	Completed	Measure changes in biomarkers of epithelial plasticity such as N-cadherin and vimentin following bone targeting radium-223 in those with mCRPC. No results posted.
	*NCT00887640*	2	Terminated	Measure percent change in N-cadherin expression at baseline and at 8 weeks following treatment with Temsirolimus in those with mCRPC refractory to treatment.

Abbreviations: ADT: androgen deprivation therapy; CCL2: monocyte chemoattractant protein-1; CDH1: cadherin-1; EZH2: enhancer of zeste homolog 2; FGF: fibroblast growth factor; HSP90: heat shock protein 90; IL6: interleukin-6; LSD1: lysine-specific demethylase 1; mCRPC: metastatic castration-resistant prostate cancer; MEK: mitogen-activated protein kinase kinase; MTD: maximum tolerated dose; NCT: national clinical trial; PRC2: polycomb repressive complex 2; PSA: prostate-specific antigen; RTK: receptor tyrosine kinase; SRC: proto-oncogene tyrosine-protein kinase; TGF-β: transforming growth factor beta.

## Data Availability

Not applicable.
